# High-Power Single-Frequency Continuous-Wave Tunable 1064/532 nm Dual-Wavelength Laser

**DOI:** 10.3390/mi16111201

**Published:** 2025-10-23

**Authors:** Weina Peng, Pixian Jin, Jing Su, Jiao Wei, Huadong Lu

**Affiliations:** 1 State Key Laboratory of Quantum Optics Technologies and Devices, Shanxi University, Taiyuan 030006, China; 201912607006@email.sxu.edu.cn (W.P.); pxjin@sxu.edu.cn (P.J.); jingsu@sxu.edu.cn (J.S.); luhuadong@sxu.edu.cn (H.L.); 2 Collaborative Innovation Center of Extreme Optics, Shanxi University, Taiyuan 030006, China

**Keywords:** all-solid-state laser, high power, single-frequency, wideband continuously tunable laser, dual-wavelength laser

## Abstract

A high-power single-frequency continuous-wave wideband continuously tunable dual-wavelength laser at 1064/532 nm is presented in this paper. Firstly, a thermally insensitive cavity containing a type-I phase-matching LiB_3_O_5_ crystal and an uncoated quartz etalon was specially designed, which provided the fundamental condition for the generation of a high-power single-frequency 1064 nm and 532 nm laser. By carefully optimizing the mode matching, the maximal output powers of 13.3 W at 1064 nm and 12.5 W at 532 nm were achieved when the pump power was 63.7 W, and the total optical–optical efficiency of 40.5% was achieved. After the transmission peak of etalon was locked to the oscillating frequency of the resonator, the continuous frequency tuning ranges of the achieved laser were as wide as 26.75 GHz at 1064 nm and 53.5 GHz at 532 nm.

## 1. Introduction

All-solid-state (ASS) single-frequency (SF) continuous-wave (CW) lasers have been widely applied in lots of advanced fields including quantum information [1,2], high-resolution spectroscopy [3,4] and so on, owing to their intrinsic advantages of high beam quality, low intensity noise and narrow linewidth. Especially, ASS SF CW dual-wavelength 1064/532 nm lasers with frequency correlation, high output power and wide continuous tuning range are the essential laser sources for the generation of tunable ultraviolet lasers used in resonance ionization mass spectrometry and the generation of non-classical states of light used in gravitational-wave detection [5,6]. In the field of atom physics [7,8], single-frequency CW tunable dual-wavelength lasers operating at 1064 nm and 532 nm can also simultaneously establish the red-detuning and blue-detuning magneto-optical traps and further satisfy the requirements of atom cooling and trapping. In the field of nonlinear spectroscopy, such as coherent anti-Stokes Raman spectroscopy (CARS), the high-power, synchronized outputs at 1064 nm and 532 nm can be used to pump a tunable optical parametric oscillator (OPO) to generate the precise pair of frequencies (pump and Stokes beams) required for high-resolution molecular vibrational imaging. For laser-based material processing, they facilitate marking, welding, and cutting of materials with distinct absorption responses at infrared and visible wavelengths, improving process flexibility and efficiency. In ophthalmic surgery, an integrated system utilizing 532 nm for retinal photocoagulation and 1064 nm for vitreolysis procedures allows for comprehensive treatment within a single device, enhancing clinical workflow and precision. Such dual-wavelength sources thus meet the demanding requirements of both scientific instrumentation and industrial and medical systems. Initially, the ultra-broad continuous frequency tuning was realized in single-frequency CW microchip lasers. Benefiting from the short cavity length of a few millimeters, its longitudinal-mode spacing was so large that it was straightforward to achieve ultra-broad continuous frequency tuning of the single-frequency laser by scanning the cavity length using the piezoelectric, electro-optic, or thermo-optic effect [9,10]. However, the short cavity length resulted in a low laser gain, which led to a low output power. In order to effectively improve the output power, a spatially structured resonator including independent cavity mirrors and gain medium was designed [11]. The etalon was inserted into the resonator for fine frequency selecting. By rotating the angle of the etalon, adjusting the thickness of the air-spaced etalon, and scanning the temperature of the etalon, the frequency tuning of the laser was successfully realized [12,13]. For example, in 2013, a single-frequency CW tunable 1064/532 nm laser with the maximal tuning range of 24 GHz was realized by scanning the temperatures of the inserted fused quartz etalon [14]. The output power of the obtained tunable laser was as high as 10.5 W. However, the response bandwidths of these tuning methods are typically in sub-kHz range. In order to overcome this problem, an electro-optic etalon was employed to replace the temperature-controlled etalon. In 2014, Xing et al. [15] designed a tuning element comprised of a PBS and an electro-optic LiNbO_3_ crystal in a single-frequency Nd:YAG laser. By changing the voltage applied to the LiNbO_3_ crystal, the frequency tuning range of 142.2 GHz was realized, and the response speed of the tuning reached the GHz level. On this basis, it was necessary to lock the transmission peak of the etalon to the oscillating frequency of the resonator before continuously changing the cavity length. In 2015, D. Radnatarov et al. [16] achieved a single-frequency tunable Nd:YVO_4_/LBO laser with the continuous tuning range of 18 GHz by employing two intra-cavity locked etalons. But this method requires an additional locking system in order to achieve continuous frequency tuning, which would increase the complexity of the frequency tuning process. Another method to realize the continuous frequency tuning is to introduce a nonlinear loss into a laser resonator. Based on these methods, the maximal tuning range of 80 GHz at the wavelength of 532 nm in Nd:YVO_4_/LBO laser was achieved [17]. Furtherly, combining the locked intracavity etalon with inserted nonlinear loss, a single-frequency continuously tunable CW dual-wavelength 1064/532 nm laser with the maximal continuous frequency tuning range of 222.4 GHz was successfully realized in 2018, which was the broadest continuous tuning range accomplished only by simply scanning the cavity length; however, the output power was only 2.1 W [18]. We have compared the various parameters of tunable lasers over the years and created a table as shown in Table 1. On this basis, in order to achieve the high-power single-frequency CW tunable 1064/532 nm dual-wavelength laser, in this paper, a thermally insensitive cavity containing a type-I phase-matching LiB_3_O_5_ (LBO) crystal and an uncoated quartz etalon was specially designed, which provided the fundamental condition for the generation of a high-power single-frequency 1064 and 532 nm laser. By carefully optimizing the mode matching, the high conversion efficiency from pump source to oscillating laser was achieved. Then on the basis of manipulating the introduced nonlinear loss, the single-longitudinal mode (SLM) operation of the generated laser was obtained. Furthermore, in order to realize the wideband continuous frequency tuning range of the generated dual-wavelength lasers, the transmission peak of etalon was locked to the oscillating frequency of the resonator. Finally, the maximal output powers of 13.3 W at 1064 nm and 12.5 W at 532 nm were achieved, and the total optical–optical efficiency was as high as 40.5%. The continuous frequency tuning ranges of the achieved high power ASS SF CW dual-wavelength lasers were as wide as 26.75 GHz at 1064 nm and 53.5 GHz at 532 nm.

## 2. Experimental Setup

**Figure 1:**
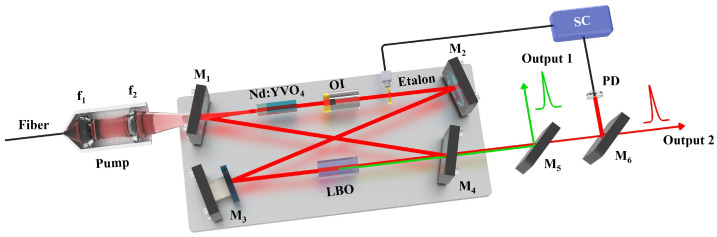
Schematic diagram of the high power wideband continuously tunable ASS SF CW dual-wavelength laser at 1064 nm and 532 nm.

**Figure 2:**
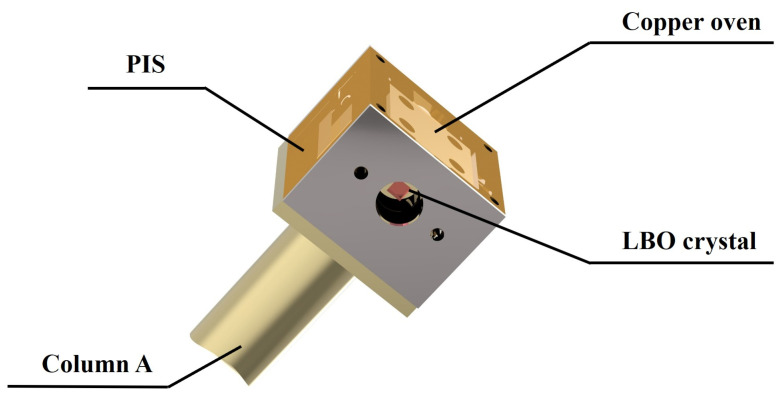
The diagram of the LBO oven device.

The schematic diagram of the designed high-power ASS SF CW wideband-tunable dual-wavelength 1064/532 nm laser was shown in Figure 1. The pump source was a fiber-coupled laser diode (LD) (DILAS M1F4S22-888.3-80C-IS30.3M6O3T3W12) with a center wavelength of 888 nm. The diameter and numerical aperture (NA) of the coupling fiber were 400 µm and 0.22, respectively. In order to achieve the best mode-matching between the pump and oscillating fundamental-wave laser modes, a telescope system consisting of two lenses *f*_1_ and *f*_2_ was designed. The focal length of *f*_1_ and *f*_2_ were 30 mm and 80 mm, respectively. The resonator adopted the classical bow-tie architecture, and it consisted of four mirrors (M_1_, M_2_, M_3_, M_4_). The input coupler mirror M_1_ was a concave-convex mirror with curvature radius of 1500 mm, and it was coated with high-transmission (HT) film at 888 nm (T_888nm_ > 95%) and high-reflection (HR) film at 1064 nm (R_1064nm_ > 99.8%). The plane convex mirror M_2_ and plane concave mirror M_3_ were both coated with HR films at 1064 nm (R_1064nm_ > 99.8%). The output coupler mirror M_4_ was a plane concave mirror and coated with HT film at 532 nm (T_532nm_ > 99.8%) and partial transmission film of 4% at 1064 nm (T_1064nm_ = 4%), so as to ensure dual-wavelength laser output while achieving the 10 W-level of optimal output power at both 1064 nm and 532 nm laser. The curvature radius of M_2_ was also 1500 mm, and the curvature radii of M_3_ and M_4_ were both 100 mm (R = −100 mm). An α-cut composite YVO_4_/Nd:YVO_4_ rod with the size of 3 mm × 3 mm × 23 mm acted as the gain medium, who included an un-doped end cap of 3 mm and an Nd-doped part of 20 mm. The concentration of the Nd-doped part was 0.8%. A wedge angle of 1.5 degree at the rear end face was designed and fabricated to purify the polarization state of the oscillating laser mode. Both end-faces of the gain medium were coated with the anti-reflection (AR) films at 888 nm and 1064 nm (R_888,1064nm_ < 0.25%), as we adopted 888 nm pumping to overcome the thermal limitations of conventional 808 nm pumping in high-power Nd:YVO_4_ lasers. The component was encapsulated in indium foil within a copper oven adhered with a thermoelectric cooler for precise temperature control. An optical isolator (OI) comprised of a half-wave plate at 1064 nm and an 8 mm terbium gallium garnet crystal (TGG) surrounded by a permanent magnet was inserted into the resonator to ensure the unidirectional operation of the laser cavity. In order to realize the dual-wavelength output of the designed laser, a type-I noncritical phase-matching LBO crystal with the size of 3 mm × 3 mm × 18 mm was employed and placed at the beam waist between the mirror M_3_ and M_4_. Both the front end-face and the rear end-face were coated with the AR films at 1064 nm and 532 nm (R_1064,532nm_ < 0.25%). The LBO crystal was placed in a copper oven, and wrapped in the polysulfone insulation shell (PIS) for maintaining a constant temperature. To detect accurate temperature, the thermistor was placed near the crystal. Its temperature was stabilized at the phase-matching point for second harmonic generation using a homemade precision thermoelectric controller (0.01 K). The entire oven, mounted on copper Column A, was housed in an integrated indium steel cavity with independent temperature control. The specific LBO oven device diagram was shown in Figure 2. At the same time, the horizontal-polarization 1064 nm and vertical-polarization 532 nm lasers leaked from the output coupler coated with polarization-insensitive were separated from each other by a dichroic mirror M_5_ which was coated with the AR film at 1064 nm (R_1064nm_ < 0.25%) and HR film at 532 nm (R_532nm_ > 99.8%). An uncoated quartz etalon (Φ 10 × 0.5 mm) with the bandwidth of 238.25 GHz at the reflectivity of R = 5.6% was inserted into the resonator and mounted on the rotation axis of a galvanometer scanner for the fine mode selection. A small part of 1064 nm laser was injected into the phase-lock system consisting of a photodetector (PD, ETX-500, JDSU Corporation, Milpitas, CA, USA) and a servo controller (SC) for locking the transmission peak of etalon to the oscillating mode of laser resonator. Once the goal was achieved, the laser frequency could be continuously tuned by scanning the voltage value loaded to the piezoelectric ceramics (PZT).

**Figure 3:**
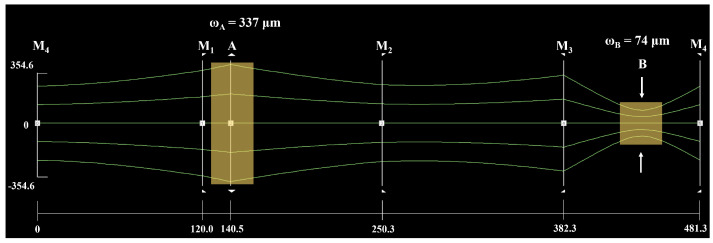
Beam transmission characteristics of the designed resonator.

For a single-frequency CW laser with the high-power operation, the mode competition was so intense that it was very difficult to achieve the SLM operation because the gain was far larger than the loss at this time [23,24]. Moreover, the multi-longitudinal-mode (MLM) oscillation or mode-hopping of the laser easily occurred with the variation of the cavity length, which would lead to the failure of continuous frequency tuning. Therefore, it was necessary to design a thermally insensitive cavity for achieving the high power wideband continuously tunable single-frequency CW dual-wavelength laser. As shown in Figure 3, the beam transmission characteristics of the designed resonator were analyzed. The total optical length of the designed cavity was 481.3 mm. The gain medium Nd:YVO_4_ was placed at point A, and the beam radius of this point was set as 337 µm to maximize the pump absorption efficiency without resulting in a detrimental thermal effect. In addition, an LBO crystal was placed at point B, where the beam waist was 74 µm, to generate a 532 nm laser and introduce appropriate nonlinear losses for achieving single-frequency operation in the CW dual-wavelength laser.

**Figure 4:**
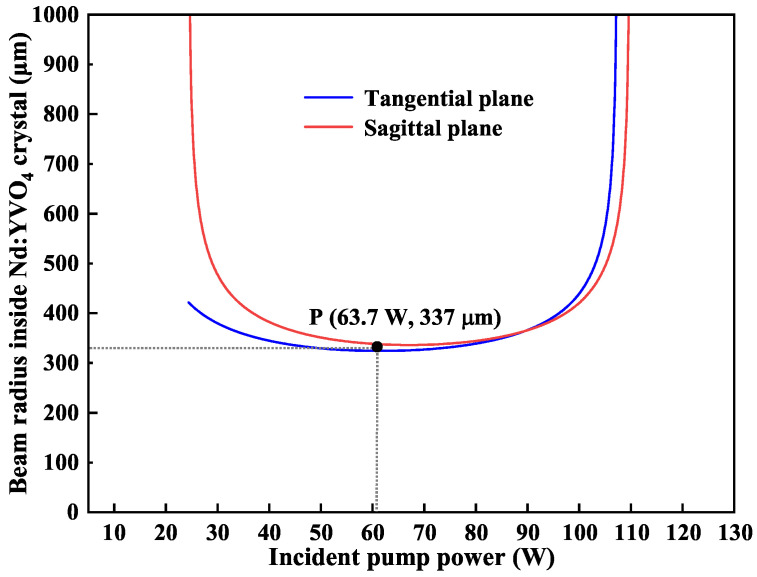
Dependencies of the mode sizes at the position of the Nd:YVO_4_ crystal on incident pump power when the incident pump power varied from 25 W to 105 W.

Although the thermal lensing effect of the gain crystal could be tolerated by the designed resonator, its thermal characteristic still had to be considered when the injected pump power exceeded the order of 10 watts. Therefore, we theoretically calculated the variations of the beam size in the Nd:YVO_4_ crystal with the increasing of the pump power, and the results were shown in Figure 4. The blue and red lines illustrated the variation trend of the beam radius at point A with respect to the incident pump power at the tangential and sagittal plane of the laser resonator, respectively. It was obvious that the resonator can operate stably when the pump power ranged from 25 W to 105 W. Specifically, when the pump power was in the range from 40 W to 90 W, both of the beam sizes at point A in the tangential and sagittal planes were approximately the same. On this basis, the beam size of the pump laser could be determined. Finally, after locking the transmission peak of the inserted etalon to the oscillating frequency of the designed resonator, the ASS SF CW tunable dual-wavelength laser was successfully achieved with output powers of 13.3 W at 1064 nm and 12.5 W at 532 nm.

## 3. Experimental Results

**Figure 5:**
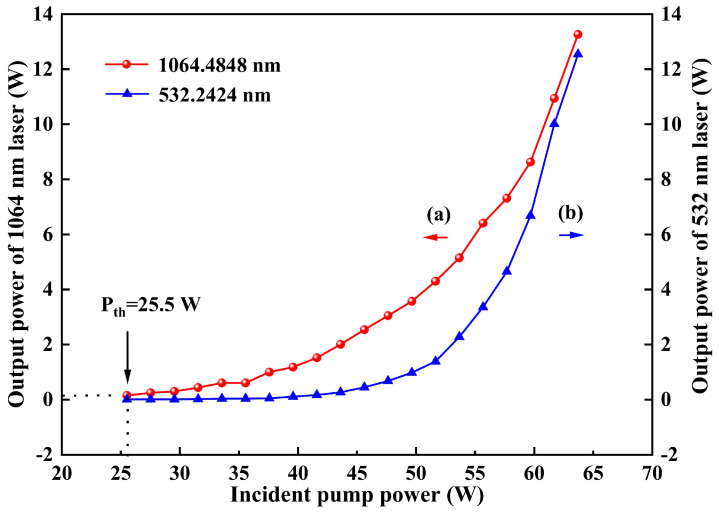
Output powers of the 1064 nm and 532 nm laser versus the incident pump power.

When the temperature of LBO was optimized to 147.18 °C, the stable single-frequency 1064 nm and 532 nm laser were obtained. Then the dependencies of the output powers of 1064 nm and 532 nm leaked from the resonator on the incident pump power were experimentally measured, which were recorded in Figure 5. It was seen that the threshold pump power was 25.5 W, and the output powers of 1064 and 532 nm kept increase to the maximal value during the entire process of increasing the pump power. Furthermore, benefiting from the accurate calculation of the thermal lensing effect of the gain crystal and the special design of the resonator above, the slope efficiency of the curve (a) increased with the continuous increase of the injected pump power. When the pump power was 63.7 W, the maximal output power of 13.3 W at 1064 nm was achieved. Meanwhile, owing to the inserted LBO crystal in the laser cavity, the single-frequency 532 nm laser with the output power of 12.5 W was simultaneously obtained, which was shown as curve (b). It is worth mentioned that the total optical–optical efficiency was up to 40.5%, which verified that the designed resonator and the mode matching between the oscillating mode and pump mode [25] were suitable for the high-power operation of laser. Notably, the output power showed no sign of saturation, indicating that further power scaling remains highly feasible.

**Figure 6:**
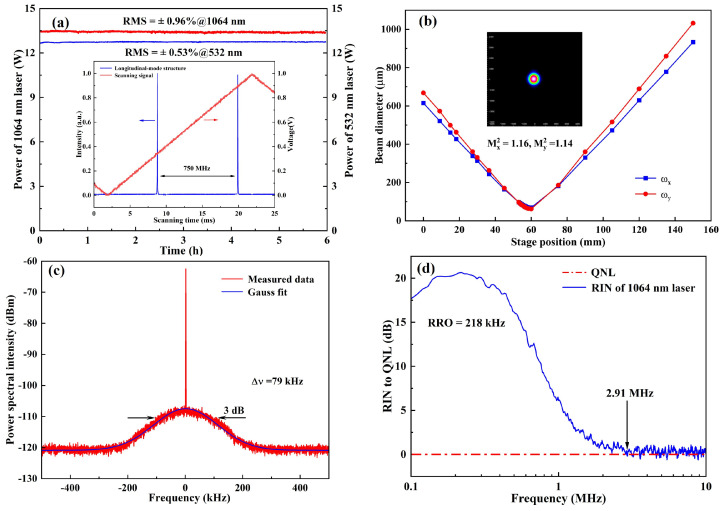
Output characteristics of generated single-frequency CW dual-wavelength laser: (**a**) power stability and longitudinal-mode structure (inset); (**b**) beam quality; (**c**) linewidth; (**d**); relative intensity noise (RIN).

The output characteristics of the designed single-frequency CW dual-wavelength laser were measured, which were shown in Figure 6. The long-term power stability of generated dual-wavelength lasers over 6 hours were recorded in Figure 6a. The calculated RMS (Root Mean Squared) power variation values of 1064 nm and 532 nm laser were 0.96% and 0.53%, respectively. Meanwhile, the longitudinal-mode structure of the 1064 nm laser was monitored by employing a homemade Fabry–Perot cavity with a free spectral range of 750 MHz and fineness of 300, and the result was depicted in the inset of Figure 6a. Then a small part of 1064 nm laser was injected into a beam quality analyzer (M2SETVIS, Thorlabs) to record its caustic curve and corresponding spatial beam profile. The results were shown in Figure 6b, and the measured beam quality factors in the X and Y directions were 1.16 and 1.14, respectively. In addition, the linewidth of the achieved 1064 nm laser was also measured by a delayed self-heterodyne interferometer based on a fiber with the path length of 25 km, and the measured result was shown in Figure 6c. It was seen that the linewidth of the single-frequency CW 1064 nm laser was as narrow as 79 kHz. Furthermore, as shown in Figure 6d, the relative intensity noise (RIN) of the single-frequency CW 1064 nm laser was measured by employing a homemade balanced homodyne detection (Yuguang Co., Ltd., Taiyuan, China). It could be seen that the resonant relaxation oscillation (RRO) frequency and the quantum noise limit (QNL) cutoff frequency of the laser were 218 kHz and 2.91 MHz. Obviously, a single-frequency CW dual-wavelength 1064/532 nm laser with good performance and power stability, single longitudinal mode, high beam quality, narrow linewidth and low relative intensity noise was achieved.

**Figure 7:**
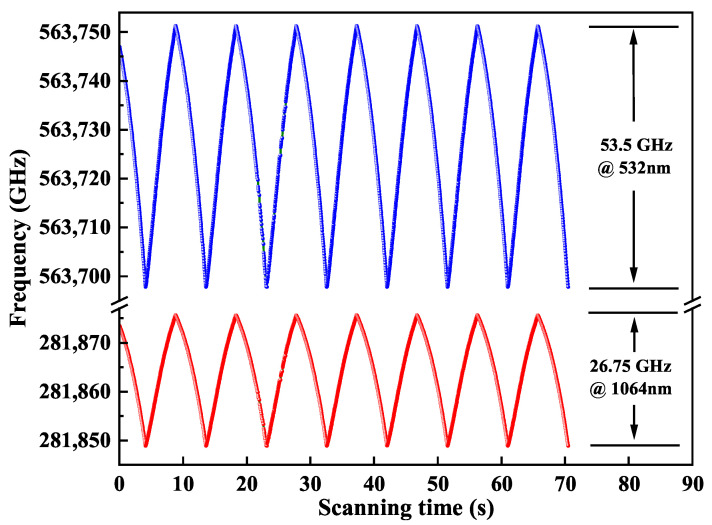
Continuously frequency tuning characteristic of the generated single-frequency CW dual-wavelength laser.

At last, a scanning signal with the frequency of 0.1 Hz was applied to the PZT for continuously changing the cavity length. A fraction of the output 1064 nm laser and 532 nm laser were separately coupled into two channels of the wavelength meter (WLM, WS6, High Finesses Laser and Electronic System) to record the frequency tuning data. The results were plotted in Figure 7. Benefiting from our designed thermally insensitive resonator with ultra-wide stable region, the wavelengths of the single-frequency CW dual-wavelength laser at 1064 nm and 532 nm can be continuously tuned from 281,848.90 GHz to 281,875.65 GHz and 563,697.80 GHz to 563,751.30 GHz, respectively. The obtained maximal continuously frequency tuning ranges were 26.75 GHz and 53.5 GHz, respectively, which can well meet the requirement of atomic physics, precision measurements or other fields.

## 4. Conclusions

In conclusion, we reported an all-solid-state single-frequency continuous-wave dual-wavelength 1064/532 nm laser, which had dual characteristics of high output power and wide tuning range. Firstly, a thermally insensitive cavity containing a type-I phase-matching LiB_3_O_5_ crystal and an uncoated quartz etalon was designed, which can stably operate with high output power. By carefully optimizing the mode matching and manipulating the introduced nonlinear loss, the single-longitudinal mode operation of the generated dual-wavelength lasers was obtained. When the pump power was 63.7 W, the maximal output powers of 13.3 W at 1064 nm and 12.5 W at 532 nm were achieved, and the optical–optical efficiency was as high as 40.5%. Furthermore, in order to realize the wideband continuous frequency tuning range of the generated dual-wavelength lasers, the transmission peak of the etalon was locked to the oscillating frequency of the resonator. By continuously scanning the cavity length, the continuous frequency tuning ranges of 26.75 GHz at 1064 nm and 53.5 GHz at 532 nm were achieved. Meanwhile, the output characteristics of the dual-wavelength laser including power stability, beam quality, linewidth and relative intensity noise were measured, which proved out that the achieved laser source with excellent performances could meet the high requirements of atom physics, precision measurements or other fields well.

## Figures and Tables

**Table 1:** Comparison of various parameters of tunable lasers over the years.

Year	Output Power	Tuning Range	Continuous Tuning
1987 [9]	1 mW@1064 nm	76.5 GHz	Yes
1992 [12]	64 mW@1064 nm	227 MHz	Yes
1997 [17]	1.3 W@532 nm	80 GHz	No
2008 [19]	480 mW@1064 nm	17.2 GHz	No
2009 [20]	25 W@1064 nm & 2.5 W@532 nm	3.2 GHz	Yes
2013 [21]	1.254 W@1064 nm	18 GHz	No
2013 [14]	10.5 W@532 nm	1.2 GHz	Yes
2014 [11]	33.7 W@1064 nm & 1.13 W@532 nm	—	—
2014 [15]	27.6 mW@1064 nm	142.2 GHz	No
2015 [16]	1.5 W@532 nm	240 GHz	Yes
2018 [18]	2.12 W@532 nm	222.4 GHz	Yes
2019 [10]	34 mW@1064 nm	27 GHz	Yes
2023 [22]	2.39 W@1080 nm & 4.18 W@540 nm	157 GHz	Yes

## Data Availability

All data reported in the paper are presented in the main text. Any other data will be provided on request.
